# Health care provider experiences in primary care memory clinics: a phenomenological study

**DOI:** 10.1186/s12875-018-0756-z

**Published:** 2018-05-19

**Authors:** Linda Sheiban, Paul Stolee, Carrie McAiney, Veronique Boscart

**Affiliations:** 1 School of Public and Health and Health Systems, University of Waterloo, Waterloo, Ontario Canada; 2 Schlegel Centre for Advancing Seniors Care, Conestoga College, Kitchener, Ontario Canada; 3 Schlegel-UW Research Institute for Aging, Kitchener, Ontario Canada; 4 Department of Psychiatry and Behavioural Neurosciences, McMaster University, Hamilton, Ontario Canada

**Keywords:** Alzheimer’s disease, Dementia, Primary health care, Gerontology, Phenomenology, Qualitative methodology, Memory clinics

## Abstract

**Background:**

There is a growing need for community-based services for persons with Alzheimer’s disease and related dementias (ADRD). Memory clinic (MC) teams in primary care settings have been established to provide care to people with ADRD. To consider wider adoption of these MC teams, insight is needed into the experiences of practitioners working in these models. The purpose of the current study is to explore the experiences of health care providers (HCPs) who work in primary care Memory Clinic (MC) teams to provide care to persons with Alzheimer’s disease and related dementias (ADRD).

**Methods:**

This study utilized a phenomenological methodology to explore experiences of 12 HCPs in two primary care MCs. Semi-structured interviews were completed with each HCP. Interviews were recorded and transcribed verbatim. Colaizzi’s steps for analyzing phenomenological data was utilized by the authors.

**Results:**

Three themes emerged from the analysis to describe HCP experiences: supporting patients and family members during ADRD diagnosis and treatment, working in a team setting, and personal and professional rewards of caring for people with ADRD and their family members.

**Conclusions:**

Findings provide insight into current practices in primary care MCs and on the motivation of HCPs working with persons with ADRD.

## Background

As the prevalence of Alzheimer’s disease and related dementias (ADRD) increases, there is a growing pressure on the healthcare system to provide care for individuals with dementia and their caregivers. Primary care is the first level of health care in which people with dementia seek and receive care (family physician clinic). However, there are concerns about the capacity of primary care [1] to address complex, chronic conditions [2]. Implementing collaborative care models for chronic conditions such as ADRD has been shown to improve quality of care, and is linked to positive health outcomes for patients [3, 4]. The diagnosis and management of patients with ADRD is a challenge in primary care [5]. A systematic review by Bradford and colleagues [6] found that ADRD is difficult to diagnose and manage in primary care settings due to several factors, including lack of proper training in distinguishing between the different types of dementia.

The Ontario College of Family Physicians developed a five-day training program to implement a primary care-based Memory Clinic (MC) model that supports multidisciplinary team approaches to diagnose and treat people with ADRD [5]. This specialized memory clinic model has been adapted to fit a primary care structure, training already-established primary care teams on adopting a memory clinic model within their current primary care practice setting [7]. Teams who have embedded this model hold MC days monthly to assess referred patients [5]. The memory clinic model in Ontario offers a multidisciplinary team training approach to better assist in the diagnosis and treatment of ADRD among health teams [8]. Team members are trained to work in this clinic, and provide comprehensive, collaborative assessments to patients who present with memory issues [5]. These clinics offer an intermediate level of care between primary and specialist care for patients who present with symptoms of ADRD. Family Health Teams (FHTs) are the most common teams to embed this model, with various healthcare providers (such as nurses, physicians, social workers and pharmacists) collaborating [5].

The effectiveness of primary care MCs and their societal impact is not yet understood. Previous studies have examined experiences of those working with people with ADRD in acute care [9] and psychiatric care [10]. Lee and colleagues [5] showed that a MC decreased the number of unnecessary referrals to specialists and physicians believed that MCs improved the quality of care they provided. In order to consider wider adoption of primary care based models, insight is needed into the experiences of practitioners working in these models.

The purpose of this study was to explore the experiences of health care providers (HCPs) who work with people with ADRD and their families. Understanding the experiences of HCPs in MCs will assist in developing strategies to enhance primary care services [11]. This work could promote understanding of HCPs’ responsibilities and practices and inform future policy.

## Methods

A phenomenological approach was employed to explore the essence of the experience of HCPs working in primary care MC teams in Ontario when caring for people with ADRD. This approach aimed to obtain a deeper understanding of the nature of the shared experience of HCPs who care for patients with ADRD in primary care memory clinics [12, 13]. These HCPs share a common experience of working in an enhanced model of primary care (memory clinic) and in a team setting to provide care for patients with ADRD. The study received ethics clearance from the University of Waterloo Office of Research Ethics in Ontario, Canada (ORE # 19001).

### Recruitment & interview protocol

Eligibility criteria for HCP participation included: 1) currently working in FHT-based primary care MC; and 2) English-speaking. Interested team members contacted the Principal Investigator (PI) to schedule an interview. Participants signed an informed consent prior to participation. Individual interviews took place in a private room within the work setting. The PI utilized a semi-structured interview guide, allowing for flexible probing of examples, thoughts and ideas.

### Data analysis

Interviews were recorded and transcribed verbatim. The authors used Colaizzi’s [14] steps to analyze the data. These steps included the following procedure. First, each participant’s transcript was reviewed by the PI by reading what they stated during their interview. This step allowed the researcher to gain a sense of their experience. Next, phrases that pertained to the phenomenon were extracted [14]. The PI then “formulated meanings” by moving beyond each statement to determine its meaning, while keeping the original transcription in mind [14]. The meanings of statements were then organized into theme clusters. Two authors compared these themes to the transcripts, validating that emerging themes kept the essence of the original statements [14]. If authors revealed differences in opinions, they reflected on the purpose of the study and engaged in several discussions to ensure agreement on the final themes [15]. Themes were then used to construct a description of the topic under investigation. Member-checking ensured themes captured the true nature of experiences.

## Results

### Background of participants

A total of 12 HCPs from two MC teams participated in the study. Participants included four nurses, two primary care physicians, two social workers, two pharmacists, one physician assistant and one occupational therapist. Participants’ experience ranged from 1 to 7 years in the MC and from 1 to 33 years in their HCP role. Interviews lasted 30 minutes. Data saturation was reached after six interviews as no new themes and ideas emerged [13]; however, the PI completed 12 interviews to ensure a full range of experiences were included. Four HCPs provided feedback as part of the member-check, assisting in validating the findings.

### Themes

Three main themes emerged from the analysis (Table 1): 1) supporting patients and family members during ADRD diagnosis and treatment, 2) working in a team setting, and 3) personal and professional rewards of caring for people with ADRD and their family members.

**Table 1:** Overview of themes and subthemes

Themes	Subthemes
Supporting patients and family members during ADRD diagnosis and treatment	*Maintaining patient dignity*
	*Balancing emotional situations and responsibilities*
Working in a team setting	*Feeling valued and connected to team members*
	*Unique health care provider experiences*
Personal and professional rewards of caring for people with ADRD and their family members	*Complexities*
	*Ongoing learning opportunities*
	*Personal and professional fulfillment*

#### Theme 1: Supporting patients and family members during ADRD diagnosis and treatment

HCPs in the MC felt they supported patients and their families through the course of the ADRD diagnosis and the care plan. They felt responsible for patients and families, and discussed the importance of supporting patients while maintaining their dignity.

##### Subtheme 1: Maintaining patient dignity

Obtaining the health and social history of patients allows HCPs to understand the patient’s identity, which assists them in maintaining a patient’s dignity. One HCP described the MC as a “soft place to land” for patients, providing them and their family the opportunity to discuss challenges and solutions with the HCPs. The HCPs take the time to listen while demonstrating a caring attitude:

“I like to think of it, like, we're a soft place to land. We can sort of help buffer whatever hell they're going through. So if they go away going ‘wow, somebody cares’ that makes it all worthwhile,” [HCP11].

HCPs described how an ADRD diagnosis affects the emotional state of patients and families and how their role consists of seeing the person behind the diagnosis:

“I think that the most challenging part would be, dancing around the dignity piece. Many people don’t want to hear the word dementia. So it’s being able to deliver the language or concerns with still maintaining who they are and the dignity behind that person,” [HCP03].

Understanding the stigma associated with an ADRD diagnosis helps HCPs to recognize and respond to these feelings and was perceived as an important role of their work in the MC.

##### Subtheme 2: Balancing emotional situations and responsibilities

One of the most difficult situations for HCPs in the MC was delivering the “bad news” of an ADRD diagnosis. When asked how one “breaks bad news”, participants talked about empathic responses and the importance of observing patients’ and families’ reactions to the diagnosis. These emotions often directed how HCPs responded to the situation:

“Sometimes, if they’re really sad or upset, then I feel that too. I feel sad for them. But some patients, they understand. So I sort of react according to how the patient reacts. If they’re OK with it, then I feel OK. If they’re really upset and sad, then I feel really upset and sad too” [HCP06].

Once a diagnosis had been made, HCPs discussed feeling responsible to help patients and their families on this emotional journey. One responsibility of the HCPs at the MC is assessing the patient’s ability to maintain their driver’s license. When asked how revoking licenses made them feel, many HCPs described feelings of sadness. HCPs understand that driving is an important part of most peoples’ lives so it is a difficult issue for them to discuss:

“Reporting licenses? Huge, huge. Like the hardest thing you have to do. People hate you, scream at you, yell at you, walk out, storm out. It's really hard for people and you hate doing it.” [HCP11].

HCPs discussed the importance of gaining all necessary information from patients and families in order to accurately diagnose ADRD. HCPs described how a limited understanding of the disease by patients and families could form a barrier to adherence, such as issues around adhering to prescribed medications and attending follow-up appointments. One HCP described the struggle of accepting decisions made by patients and families:

“Once in a while, you get somebody who just says: “I don't want the pills. I don't want anything”. And you know they have things really wrong with them that you could help with” [HCP09].

All HCPs described how they try to understand the journey of a patient and their family, and their role of helping people make decisions that could enhance quality of life. HCPs saw this as their main responsibility at the MC:

“I just love the fact that so many people surround this family and form a safety net. So I think that's really rewarding and you can see the difference that it makes and how much people appreciate that kind of care” [HCP10].

HCPs aim to provide various levels of support to patients and families when giving difficult news. HCPs talked about using their expertise to guide people on the ADRD journey.

#### Theme 2: Working in a team setting

The second theme describes HCPs’ experiences working as a team within the MC. HCPs spoke about their strong connection with their colleagues, feeling part of a “family”. HCPs explained the unique experience of working at the MC versus other health teams, specifically other FHTs. Two subthemes describe *feeling valued and connected to team members* as well as having *unique HCP experiences*.

##### Subtheme 1: Feeling valued and connected to team members

HCPs described the value of individual roles in the MC. They believed that having a variety of disciplines on the team enhanced opportunities for comprehensive and multi-faceted care:

“The disciplines all bring their own lens and perspectives. Bringing that to the table and having a really open, fulsome discussion is a much richer, and fulsome assessment than any individual physician can have in their office visit” [HCP08].

HCPs discussed their professional experience and contributions to the team. One HCP described a situation where the team brought different perspectives and collaborated to develop an individualized care plan for a patient:

“[the other HCPs are] quite respectful if I see something differently. There was a client with some behaviours. They were going to look at medication. I was trying to figure out why that behaviour was happening instead of just medicating the behaviour. And they were quite open to that” [HCP04].

Another HCP talked about being valued as a member of the team:

“I don’t think I want the team to go ahead without me. It’s part of a family and you want to be involved... I feel like there is a significant role there for [me] and that without it, there would be something missing” [HCP02].

Many HCPs described their team as a “family,” working together to care for patients with ADRD and families. HCPs felt comfortable stating their opinions during consultations. Yet, inviting different perspectives from the team could also lead to conflicting opinions. One HCP described:

“I think you could call it a challenge, like someone thinks the complete opposite of what I'm thinking. I think that's what we're looking for, the different perspectives” [HCP05].

HCPs saw benefits in gaining different perspectives, expressing opinions, and, at times, disagreeing with each other. Perceiving the team as a family allowed HCPs to speak freely and share perspectives: “…We hear everybody’s opinion, work it out and by the time everybody leaves the room [they’ve] all kind of come to an agreement” [HCP02].

A well-connected team brings advantages to caring for people with ADRD, as patients and families benefit from all HCPs’ expertise, insights and opinions.

##### Subtheme 2: Unique health care provider experiences

HCPs discussed the unique experience of working in the MC. The “tight-knit” team and the clinic set-up allowed for face-to-face consultations with team members:

“[The MC is] truly a team whereas in a Family Health Team, we have different practitioners working side by side - often still in a bit of a silo in terms of doing our own thing.” [HCP02].

HCPs described the MC team as a “little family within a big family” [HCP03]. HCPs explained how the MC set-up allows for different HCPs to see the patient during the same appointment:

“I think [the MC is] a true team environment, whereas in all the other settings, you're kind of doing the dual role, but we're not actually a team—we’re more kind of different cogs in a machine” [HCP06].

HCPs described that the time spent with the patient is unique in primary care. Having more time to consult with the patient and team members was seen as beneficial:

“I just found it so hard in the regular structure of practice—that 10-minute office visit. With these seniors, 10 minutes are to deal with the most acute problem, and memory problems are not acute until they hit crisis. So that all just got pushed back and then, nothing would be done” [HCP08].

HCPs perceived extra time to assess the patient as necessary to provide an accurate ADRD diagnosis, and positively impacts care for patients and families:

“I really like that we get to see patients consistently. When people are diagnosed with dementia, you're told and you never see that doctor again. I feel like that we are consistently seeing people at follow-up” [HCP07].

HCPs expressed that extra time provides patients and families with an opportunity to feel connected to and comfortable with the care team. Follow-up with patients is a benefit of the MC structure and supports HCPs to provide continuous care:

“We always get the same nurses to test each person over time and they have a really good sense if the person is declining. And sometimes, it's very subtle. There is just something about him that is not right. Even though his cognitive test scores seem the same. There's so many aspects to how a person functions. Those pieces of information will get lost if there wasn't this real time discussion” [HCP08].

The MC structure provides HCPs the opportunity to maintain care continuity with patients and families so they can notice changes over time. This structure also allows HCPs to connect with patients and families at different points in the ADRD journey:

“Working with the elderly, you're working at all parts of the system—right from primary care to long-term care. You meet [people] at this part, but you might meet them again at this end. So we are helping them get to the next place that they need to be…” [HCP01].

Although the MC structure allows HCPs to deliver resources to patients and families, navigating the healthcare system is a difficult task, even for HCPs. Connecting patients with the appropriate resources was challenging. One HCP explained the difficulty of finding resources for those whose drivers’ licenses were revoked:

“ [A challenge is] trying to figure what resources are available, and how we get those services in place. Specifically around driving. It’s a huge cost to the family and there’s nothing really local.” [HCP05].

Another HCP described being frustrated with the waitlists for long-term care. “The waitlists for a lot of things - in long-term care, sometimes people have to be placed on waitlists for that, it’s frustrating,” [HCP11]. Contrastingly, HCPs described the benefit of linking with community partners to assist patients and family members:

“Those community partners that we’ve connected with—the day program, the Alzheimer’s Society, and other community networks. There’s been some amazing links” [HCP03].

The MC structure allowed HCPs within that “little family” to support the “bigger families.” HCPs described the benefit this structure has for community physicians:

“We’re actually there to support the physicians in our community. We can be someone that our FHT can lean on. I like that feeling” [HCP03].

HCPs in the MC worked within a larger realm of care to provide ongoing support to those with ADRD. Although navigating resources was challenging, community partners allowed HCPs to provide additional support and resources to patients.

#### Theme 3: Personal and professional rewards of caring for people with ADRD and their family members

The final theme describes how HCPs felt they have grown personally and professionally through their work on the MC team. HCPs emphasized how the complexity of ADRD creates learning opportunities, as “no two patients are the same” [HCP01]. HCPs explained how collaborating to diagnose and support patients was like solving a puzzle, with each team member adding pieces of information.

##### Subtheme 1: Complexities

When asked about their motivations for working with the ADRD population, HCPs described finding complexities intriguing, and preferred to delve into patients’ past and current medical and social conditions in order to better understand the patient and develop a treatment plan. One HCP described the unique aspects of older patients:

“What we do in geriatrics is so much more different than, what I call the, single disease state of ‘whoa, we have your blood pressure at a great number’ and that's easy to do, relatively speaking. That's why I love what I do, because it's much more complicated—a lot of other pieces to consider—and to try to figure them out in a way that makes sense for the patient.” [HCP09].

Greater patient information was perceived as important for HCPs caring for those with ADRD. The MC allowed for more time to be spent with patients, so HCPs could collaboratively unravel the complexities and develop proper treatment strategies. One HCP described:

“…I have two new patients on Wednesday, they’re two-hour appointments. And it’s a long process. I think we live in a society where everything is quick and easy. This isn’t fast... A lot of people [say]: ‘I don’t want to deal with that. I want pretty medicine’, but they don’t know what they’re missing…” [HCP11].

Other HCPs described working in the MC as “solving a puzzle”, as there are many complex components that must fit together in order to provide quality care. One HCP described the experience of solving a mystery in the game Clue™:

“It’s like a game of Clue™. It’s like we’re all part of this detective game, we’re all doing a piece to put in” [HCP01].

HCPs also discussed the importance of using a team approach to solve these “puzzles”:

“We’re truly kind of all coming together, putting everything together… like putting a puzzle together. We’re all in the room with all the pieces, versus you all have a quarter of the puzzle… And you’re in your own offices trying to put these pieces together, it doesn’t work, unless you all come together in the same space” [HCP02].

HCPs relied on the team, patients and families to collect all necessary pieces of information, “a triangulation of evidence” [HCP08], to properly diagnose and support patients with ADRD. Working in the MC allowed HCPs to collaborate and develop innovative ways of supporting patients and families. A HCP described a team approach to managing one patient’s hallucinations:

“He has Lewy Body, and he sees these creatures that come out of the wall at night. And we needed to help them … We came up with, let’s put a spot light in his room at night, and put it towards that part of the wall [where the creatures come though]. And let’s add a fan, so the light goes on and the fan blows them [creatures] away. So he could sleep through the night. We come up with strategies and roles and supportive ideas…” [HCP11].

This HCP described helping patients with complexities and taking the time to solve the “puzzles” in collaboration with colleagues, patients and families.

##### Subtheme 2: Ongoing learning opportunities

HCPs felt rewarded working with the ADRD population as it allowed for professional growth and enhanced knowledge in dementia and patient complexities. The MC was described as a setting with learning opportunities that other care environments may not offer:

“We've learned from each other. So when I'm in my office working solo, the things that I now think of, I wouldn't have thought of before…” [HCP02].

HCPs developed other care strategies through working within a MC team. Interprofessional collaboration allowed HCPs to understand how other disciplines respond to situations. HCPs described these unique opportunities:

“I learn something from every one of them, almost every week… about what they do, what their role is, or how they approach patients and families. Everybody has their own way of saying it or maybe an expression that they use to get them to understand.” [HCP12].

The training for MCs increased HCP confidence in their knowledge and understanding of ADRD. One HCP described a previous experience regarding the challenges of a dementia diagnosis:

“…prior to working here, I probably did come across people with Alzheimer’s, but I wouldn’t have recognized that. I had zero training other than what we touched upon in school. I would just think: they’re old or they’re difficult. So it wasn’t actually until I started working here that I even knew anything about it at all” [HCP12].

##### Subtheme 3: Personal and professional fulfillment

Personal and professional fulfillment emerged as a distinctive part of HCPs’ experiences working in the MC. One HCP described knowing they were “making a difference” was a major motivation to remain at the MC:

“…even though at the end of a memory clinic day, I’m utterly exhausted… we know we’ve done good work, we’ve made a difference. And, that’s my motivation—knowing that we’re making a difference for the good” [HCP03].

Another HCP provided an example of making a difference in the MC:

“We've had a patient who was psychotic and had hallucinations of infidelity… great deal of stress for everybody. So with a little bit of fiddling around with one medication, after a couple of weeks, day and night difference. It's one of those few things where you can just say ‘wow you've made a difference.’ And so it's always rewarding,” [HCP09].

Other HCPs described the drive behind their motivation:

“If you didn't have a passion for what you were doing, I don't think you could do [it], because it's a really tough clinic. But the passion keeps me going. And there's such a need. These people need help and a lot of people don't want to help them, so that's what keeps me going.” [HCP11].

HCPs explained that only those committed to this work find it meaningful. “Just knowing it’s effective and good work” [HCP07] is a motivator for HCPs to keep them at the MC. In addition, receiving positive feedback from patients and families was rewarding.

When asked why they choose to provide care to seniors, HCPs often alluded to previous exposure to gerontology and a growing interest, and others discussed influential mentors. HCPs described mentors as individuals who understood the demanding, yet rewarding nature of working with patients with ADRD, and who assisted them in shaping their career path.

HCPs believed that their work was a “good fit” with their personality traits and professional backgrounds, and that working with the elderly takes a special kind of person: “I feel motivated to work with this population because it’s something that takes a special person to work with the geriatric population…” [HCP05]. Another HCP stated:

“I always say, “I’d do this for free if I had to”. Because I love it - but why do I love it? I love it because it just suits the way I think” [HCP01].

HCPs are strongly motivated by the team, previous experiences, mentors, and positive feedback from patients and families.

## Discussion

Findings of this study provide insight into HCPs’ experiences supporting patients and families in MC teams. HCPs take pride in their work in MCs. HCPs described how their roles fit well within the clinic team, and the difference working in the FHT versus the “tight-knit” MC, particularly opportunities for strong assessments and rich professional experiences.

HCPs described themselves as a source of support for patients and families. Maintaining patients’ dignity and identity is important for HCPs, which is consistent with other research [16, 17]. Organizations such as the Alzheimer Society of Canada stress the importance of respecting persons with ADRD by reaffirming their sense of self [18].

During MC appointments, patients are asked to bring a family member or friend. HCPs described these individuals as key players to absorb information that may be missed by MC patients. Strong relationships between patients and family members or friends enable better dementia care as families/friends may notice changes that clinicians may not [19]. Contrastingly, denial and misinformed families could prohibit an accurate ADRD diagnosis and treatment plan for patients [19]. This study confirms the essential role of families in ADRD care.

Negative reactions from patients and family during a diagnosis such as ADRD can sometimes evoke emotions among HCPs, such guilt and may lead to job stress and burnout [20]. In a systematic review by Wilkinson and colleagues [21] there was a negative relationship found between burnout and empathy amongst HCPs. Thus, obtaining necessary information in order to accurately diagnose ADRD is important to support HCPs’ job quality. One of the challenges faced by HCPs in MCs was the responsibility of revoking driver licenses. It is important to recognize that a diagnosis of ADRD may pose risks to those who are driving [22]. This study found that not only did the physicians feel responsible for revoking licenses, but other team members felt they have an important role in ensuring patient safety. Goldman and colleagues [23] identify shared responsibility as an important aspect of efficient teamwork. Although HCPs stressed the provision of alternate resources for patients after revoking a license, they expressed frustrations with navigating the system.

HCPs felt they were connected and worked well together to support patients with ADRD. Constant connectedness, close working relationships and communication within teams enhance care provided [24]. A disconnect and lack of integration among team members can potentially impede care for patients [24]. Although HCPs acknowledged these potential issues, they valued each other’s opinions, explaining that clear communication led to effective collaboration. Clear communication was the main theme in HCP interviews by Moore and colleagues [11], who investigated perspectives of physicians and nurses in a primary care program for seniors. Despite the unique context of the Ontario, Canadian funding of these primary care memory clinics, in the increase of dementia diagnosis, the importance of care team approaches can be found in European studies [25].

HCPs discussed their interest in geriatrics. Low interest in geriatrics may stem from the lack of exposure and acquired knowledge during education [26, 27]. HCPs in this study identified past experiences in gerontology as motivators to pursue their careers. HCPs preferred working in a team and discussed learning opportunities when addressing care complexities.

HCPs described the opportunity for personal and professional growth in the MC. Uncertainties in diagnosing ADRD are common among primary care physicians [19, 28]. In this study, HCPs feel comfortable with the diagnosis process, are motivated by their team, and see the importance and value of each role and the contributions they bring to the team. Goldman and colleagues [23] stressed the importance of understanding the responsibility of each role in effectively working in a health team setting. Moore and colleagues [11] found that lack of role clarity hindered effective communication between team members. HCPs in this study felt their role was clearly defined and valued by others.

### Strengths and limitations

Several strengths and limitations should be discussed. Contacting the MC Directors may have created pressure for HCPs to participate; yet, HCP participation was voluntary and participation information was not shared with Directors. Furthermore, transferability of the findings may be limited, as participating teams may have been high-functioning. Though these teams have been collaborating for several years, experience varied among participants; however, this did not seem to affect participants’ perspectives. Saturation was reached halfway through data collection, but 12 interviews were completed to ensure representative data. The researchers attempted to increase transferability by including one rural and one urban MC. In addition, participants may not have been comfortable enough to speak to the challenges of working in team settings to provide care for persons with ADRD. The PI attempted to lessen this bias by reminding participants of the confidentiality measures being taken. Lastly, interviews completed were shorter in length than typical phenomenological interviews [13], due to time constraints. However, interviews allowed the researchers to ask all questions and use probes to gain further insight.

## Conclusions

This study provides insight into HCPs’ experiences in primary care MCs. Understanding HCPs’ motivations to work with complex populations may assist in motivating future HCPs. Knowledge of the complexity of ADRD is important to ensure proper care and services. The study also provides support for team-based approaches in primary care.

## Abbreviations

ADRD: Alzheimer’s disease and related dementias
HCP: Health care providers
MC: memory clinic
